# Activity-Modulating Monoclonal Antibodies to the Human Serine Protease HtrA3 Provide Novel Insights into Regulating HtrA Proteolytic Activities

**DOI:** 10.1371/journal.pone.0108235

**Published:** 2014-09-23

**Authors:** Harmeet Singh, Tracy L. Nero, Yao Wang, Michael W. Parker, Guiying Nie

**Affiliations:** 1 MIMR-PHI Institute of Medical Research, Clayton, Victoria, Australia; 2 Monash University, Clayton, Victoria, Australia; 3 ACRF Rational Drug Discovery Centre, St Vincent’s Institute of Medical Research, Fitzroy, Victoria, Australia; 4 Department of Biochemistry and Molecular Biology, Bio21 Molecular Science and Biotechnology Institute, the University of Melbourne, Parkville, Victoria, Australia; Oxford University, United Kingdom

## Abstract

Mammalian HtrA (high temperature requirement factor A) proteases, comprising 4 multi-domain members HtrA1-4, play important roles in a number of normal cellular processes as well as pathological conditions such as cancer, arthritis, neurodegenerative diseases and pregnancy disorders. However, how HtrA activities are regulated is not well understood, and to date no inhibitors specific to individual HtrA proteins have been identified. Here we investigated five HtrA3 monoclonal antibodies (mAbs) that we have previously produced, and demonstrated that two of them regulated HtrA3 activity in an opposing fashion: one inhibited while the other stimulated. The inhibitory mAb also blocked HtrA3 activity in trophoblast cells and enhanced migration and invasion, confirming its potential *in vivo* utility. To understand how the binding of these mAbs modulated HtrA3 protease activity, their epitopes were visualized in relation to a 3-dimensional HtrA3 homology model. This model suggests that the inhibitory HtrA3 mAb blocks substrate access to the protease catalytic site, whereas the stimulatory mAb may bind to the PDZ domain alone or in combination with the N-terminal and protease domains. Since HtrA1, HtrA3 and HtrA4 share identical domain organization, our results establish important foundations for developing potential therapeutics to target these HtrA proteins specifically for the treatment of a number of diseases, including cancer and pregnancy disorders.

## Introduction

High-temperature requirement (HtrA) proteins belong to a unique family of oligomeric serine proteases that are conserved from prokaryotes to humans [1]. HtrAs in general are involved in protein quality control through sensing protein folding stress and regulating signal transduction cascades [2]. In contrast to other quality control proteases, some HtrAs such as DegP of *Escherichia coli* also exhibit a chaperone function to stabilize specific proteins [3].

There are four human HtrAs in the genome: HtrA1, HtrA2, HtrA3 and HtrA4 [2], [4]–[9]. These HtrAs play important roles in cell growth, apoptosis, invasion and inflammation; they also control cell fate via regulating protein quality control [2]. The altered expression of human HtrAs is associated with a number of diseases, including cancer, arthritis, neurodegenerative and neuromuscular disorders, age-related macular degeneration, and the pregnancy-specific disease preeclampsia [1], [10]–[16].

HtrA proteases are comprised of a serine protease domain and one or more C-terminal protein-protein interaction domains [1]. They usually form higher order oligomers ranging from ∼100 kDa up to 1.2 MDa [17]–[21]. All HtrA proteases share a common trimeric pyramidal architecture, where each monomer comprises two or three major domains, and exhibit a similar mechanism of activation [22]. Alteration of the N-terminal structural organization leads to functional diversity amongst the HtrA members and amino acid sequence variation gives rise to their substrate specificity [23]–[26].

**Figure 1:**
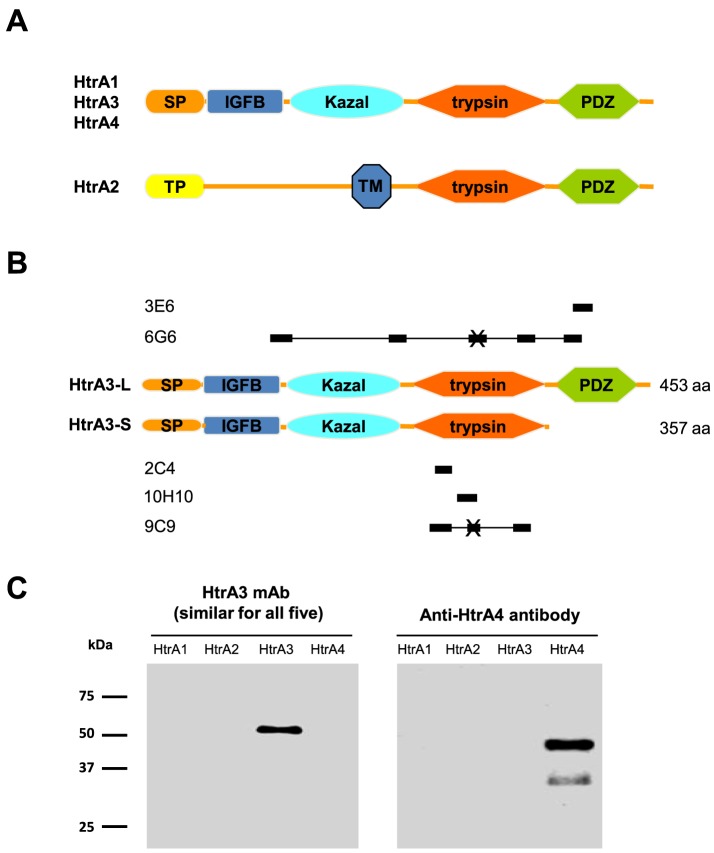
Schematic representation of HtrA3 domain organization, mAb epitope locations and confirmation of mAbs specificity. (A) The domain structure of HtrA1, HtrA3, HtrA4 and HtrA2. (B) The domain structure of HtrA3-L and HtrA3-S. The solid bars above or below the protein domains denote the locations of epitope residues of each mAb identified by the linear peptide library mapping assay. “X” indicates a peptide deemed likely to be a false positive. SP, signal peptide; IGFB, IGF-binding domain; Kazal, Kazal-type S protease inhibitor domain; trypsin, trypsin-like serine protease domain; PDZ, PDZ domain; TM, transmembrane; TP, transient peptide. (C) An equal amount (50 ng) of recombinant human HtrA proteins HtrA1, HtrA2, HtrA3 (HtrA3-L-S305A) and HtrA4 were separated on reducing 12% SDS-PAGE gels and analyzed by Western blot with HtrA3 mAbs (3E6, 6G6, 2C4, 10H10 and 9C9), and an HtrA4-specific antibody.

HtrA1, HtrA3 and HtrA4 share an identical domain organization, suggesting that they may have similar functions, but they display different tissue expression patterns [7], [9]. In contrast, HtrA2 has a completely different N-terminal domain architecture (Figure 1A) [7]. Recent structural and biochemical studies have demonstrated that HtrA1 exists as a trimer and that a conformational change induced by substrate binding is required to stimulate its proteolytic activity [27], [28]. It has also been reported for HtrA1 that neither its N-terminal region [an insulin-like growth factor binding domain (IGFB) and a Kazal-type protease inhibitor domain (hereafter referred to as the Kazal domain)] nor the C-terminal postsynaptic density protein 95-Discs large-Zona occuldens 1 (PDZ) domain has any involvement in the protease activity [27]–[29].

Mammalian HtrA3 was initially identified in the developing placenta as a serine protease associated with pregnancy both in the mouse and human [7], [30]–[32]. Two HtrA3 isoforms [long (HtrA3-L) and short (HtrA3-S)], due to alternative mRNA splicing, have been identified in the human placenta [7]. HtrA3-L has four distinct domains including an IGFB, Kazal, trypsin-like serine protease (referred to as the protease domain) and PDZ domain (responsible for protein-protein interaction) [2], [7] (Figure 1B). HtrA3-S and HtrA3-L are identical except HtrA3-S lacks the PDZ domain (Figure 1B) [7].

HtrA3 negatively regulates trophoblast invasion during placental development [33], [34] and abnormal levels of HtrA3 during early pregnancy in women are associated with risks of developing preeclampsia (a severe pregnancy-specific disorder) [15], [35]. HtrA3 is also down-regulated in a number of cancers (eg. ovary, endometrium and lung) and has been suggested to inhibit transforming growth factor-β signaling [10], [36]–[40].

To gain insight into the functional importance of HtrA3, we recently generated five HtrA3 monoclonal antibodies (mAbs), mapped their linear epitopes and established the specificity of these mAbs against HtrA1 and HtrA2 [41]. In this current study, we further establish the specificity of these HtrA3 mAbs against human HtrA4 (the fourth member of the HtrA family), and also investigate the capacity of these mAbs to modulate HtrA3 activity and their modes of action. We show that one of these mAbs inhibits whereas another stimulates HtrA3 activity with high specificity. We also demonstrate that the inhibitory mAb blocks HtrA3 activity in placental trophoblast cells to enhance migration and invasion, demonstrating its potential utility *in vivo*. To understand how the binding of these mAbs may modulate HtrA3 activity, we constructed a 3-dimensional (3D) HtrA3-L (hereafter referred to as HtrA3) homology model using HtrA1 small angle X-ray scattering (SAXS) data and mapped the location of the mAb epitopes onto the homology model. The inhibitory mAb appears to block substrate access to the HtrA3 protease catalytic site; whereas the stimulatory mAb may bind to either the PDZ domain alone or in combination with the N-terminal and protease domains. These results lay the foundation for developing therapeutics to specifically target HtrA3. Furthermore, since mammalian HtrA3 shares a similar domain organization with HtrA1 and HtrA4, our results have important implications in targeting these HtrA proteases.

## Materials and Methods

### Recombinant human HtrA proteins

C-terminally His-tagged full length recombinant (r) human HtrA1 (amino acids 1–480, Accession # Q92743, Cat # 30600102), HtrA3 (amino acids 1–453, Accession # P83110, Cat # 30600503) and HtrA4 (amino acids 1–476, Accession # P83105, Cat # 30600403) (all produced in insect cells) were obtained from ProteaImmun GmbH (Berlin, Germany), while mature HtrA2 protein (amino acids 134–458, Accession # O43464, Cat # 1458-HT, *Escherichia coli* origin) was from R&D Systems (Minneapolis, MN, USA).

### Mouse monoclonal antibody production

HtrA3 mAbs 3E6, 6G6, 10H10 and 9C9 were produced against catalytically inactive HtrA3-L-S305A, constructed by mutating the catalytic site serine residue 305 to alanine [42]; mAb 2C4 was raised against the synthetic peptide TIKIHPKKKL (corresponding to residues 230–239 in both HtrA3-L and HtrA3-S) [41]. The linear epitopes of these mAbs were determined by screening a custom-synthesized peptide library (PepSet, Mimotopes) [41].

### Cell culture

The HTR8/SVneo (HTR8) cell line was derived from primary explants cultures of human first trimester placentas (8–10 wk gestation) and immortalized with SV40 virus [43]. The HTR8 trophoblast cells, kindly provided by Dr C.H. Graham (Queen’s University, Kingston, ON, Canada), were cultured at 37°C as previously described [34].

### Western blot

Human rHtrA1, rHtrA2, rHtrA3 and rHtrA4 proteins (50 ng) were analyzed using standard Western blot (12% reducing SDS-PAGE and PVDF membrane). Primary antibodies included HtrA3 mAbs (50 µg/ml final concentration) and an anti-HtrA4 antibody (200 ng/ml final concentration, affinity-purified rabbit polyclonal, Abcam, Cambridge, UK). The membranes were incubated overnight at 4°C with primary antibodies and probed for 1 hour at room temperature with the following secondary conjugates: rabbit anti-mouse IgG HRP (1∶5000, Cell Signaling, Beverley, MA, USA) or goat anti-rabbit IgG HRP (1∶5000, DAKO, Carpinteria, CA, USA). Bands were visualized with Pierce ECL Western Blotting Substrate (Thermo Fisher Scientific, Rockford, IL, USA) and ChemiDoc MP Imaging system (Bio-Rad, Hercules, CA, USA).

### *In vitro* protease activity assay

The protease activity of HtrA3 was determined by the cleavage of a custom-made fluorescence-quenched peptide substrate H2-Opt [Mca-IRRVSYSF(Dnp)KK, synthesized by GL Biochem Ltd., Shanghai China) as previously described [27], with minor modifications.

In brief, the activity was determined in a final 50 µl reaction in half-area 96-well clear-bottomed black plates (Sigma). Firstly, a 40 µl reaction mixture containing a final concentration of 1.25 µM rHtrA3 was prepared in 50 mM Tris-HCl (pH 8.0) containing 200 mM NaCl and 0.25% CHAPS. The fluorescence-quenched peptide substrate (10 µl, final concentration 2.5 µM) was then added, and the plates were incubated at 37°C for 30 min during which the real-time kinetic progression of substrate cleavage (increase in fluorescence signal) was monitored every 15 sec at 340 nm/405 nm (Wallac, Victor 2 spectrophotometer, Perkin Elmer, MA). The rate of substrate cleavage (fluorescence increase/min) was calculated from the initial 10 min linear phase of the kinetic progression curve and used as the activity unit. In every assay, the incubation buffer was used as a blank and other controls included PBS substitution for rHtrA3.

To determine the modulatory activity of HtrA3 mAbs on the proteolytic activity of rHtrA3, the *in vitro* activity assay was performed in the presence of HtrA3 mAbs. Firstly, a 40 µl reaction mixture containing rHtrA3 protein (final concentration 1.25 µM), 5 µl HtrA3 mAbs (3E6, 6G6, 2C4, 10H10 or 2C4; final concentration 20 µg/ml) or control mAb (a non-HtrA3 mAb produced/purified similarly to HtrA3 mAbs, also at final concentration of 20 µg/ml) or buffer only, was prepared in 50 mM Tris-HCl (pH 8.0) containing 200 mM NaCl and 0.25% CHAPS. This mixture was incubated for 1 hour at 37°C, then the fluorescence-quenched peptide substrate (final concentration 2.5 µM) was added and the plates were incubated at 37°C for 30 min to monitor the real-time kinetic progression of substrate cleavage, as described above. In every assay, the incubation buffer was used as a blank and other controls included PBS substitution for HtrA3 protein and control mAb replacing HtrA3 mAbs. Additional control included 6G6 mAb only.

The dose-dependency of the inhibitory (10H10) and stimulatory (6G6) HtrA3 mAbs was further tested at a final concentration of 4 and 20 µg/ml. Furthermore, to test whether HtrA3 following 6G6 stimulation could be inhibited by 10H10, rHtrA3 was first incubated with 6G6 (20 µg/ml) for 1 hour at 37°C, then 10H10 (4 or 20 µg/ml) was added and the reaction was incubated for a further 1 hour at 37°C, before the addition of the fluorescence-quenched peptide substrate. To test if rHtrA3 following 10H10 inhibition could be activated by 6G6, rHtrA3 was first incubated with 10H10 (20 µg/ml) for 1 hour at 37°C, then 6G6 (4 or 20 µg/ml) was added and the reaction was incubated for a further 1 hour at 37°C, before the addition of the fluorescence-quenched peptide substrate. Three independent experiments were performed for each condition. Data were expressed as fold changes in the rate of substrate cleavage relative to control.

### Trophoblast cell migration and invasion in the presence of HtrA3 activity-modulating mAbs

To determine the effects of HtrA3 activity-modulating mAbs on cellular processes, real-time monitoring of migration and invasion of trophoblast HTR8 cells was carried out in the presence or absence of HtrA3 mAbs using xCELLigence, RTCA DP instrument (Roche Diagnostics GmbH, Germany) that was placed in a humidified incubator and maintained at 37°C with 95% air/5% CO_2_. For proliferation, growth curves were constructed using 16-well plates (E-plate 16, Roche Diagnostics GmbH). Briefly, HTR8 cells were seeded at 40,000/well in medium containing 1% FCS and monitored once every 2 min for 40 min and then once every hour. Following cell adhesion, HtrA3 mAbs (10H10 or 6G6, final concentration; 5 µg/ml) were added, and the plates were then monitored once every 15 min for 2 hours, then once every hour.

Cell migration and invasion were assessed using specially designed 16-well plates (CIM-plate 16, Roche Diagnostics GmbH) with 8-mm pores. These plates are similar to conventional transwell with the micro-electrodes located on the underside of the membrane of the upper chamber. To measure cell invasion, the upper surface of the transwell was coated with growth factor reduced Matrigel (BD BioSciences, Bedford, MA USA; 1∶10 diluted in serum free media). HTR8 cells were incubated with HtrA3 inhibitory (10H10) or stimulatory (6G6) mAbs each at 5 µg/ml for 20 min at 37°C prior to seeding into the upper chamber at 40,000/well in medium containing 1% FCS. In the lower chamber, the media containing 5% FCS was added as a chemo-attractant. Controls included untreated and control-IgG-treated HTR8 cells on culture inserts. The plates were monitored every 2 min for 40 min, then once every 15 min. Data analysis was carried out using the RTCA Software v1.2. Each migration and invasion assay was repeated three times and data were expressed as percent changes (± SEM) relative to untreated control.

### Construction of the HtrA3 trimer homology model

**Figure 2:**
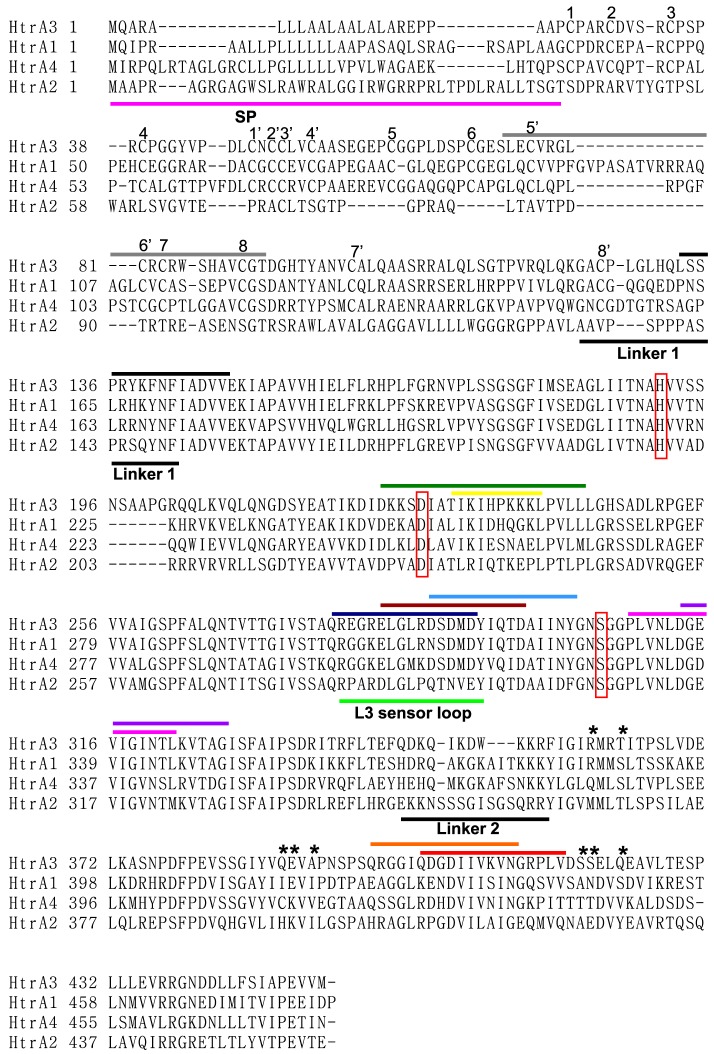
HtrA3 mAb epitope regions and sequence comparison within the human HtrA family members. The HtrA3 signal peptide (SP, residues 1–23) is defined by the pink underline. The eight disulfide bridges in the HtrA1 N-terminal domain are identified by the paired numbers (1 and 1’; 2 and 2’ etc) above the Cys residues involved. These 8 disulfide bridges appear to be conserved in both HtrA3 and HtrA4. The location of the flexible linker separating the N-terminal domain from the protease domain is indicated by the black underline labeled Linker 1; the flexible linker connecting the protease domain to the C-terminal PDZ domain is labeled Linker 2. The catalytic triad Ser-His-Asp residues are enclosed in red boxes. The location of the protease L3 sensor loop is indicated by the green underline. The black asterisks denote the HtrA3 PDZ domain residues involved in protein/peptide interactions. The location of the HtrA3 mAb epitopes are indicated by the colored lines above the HtrA3 sequence: residues 73–92 (dark grey, mAb 6G6), residues 133–147 (black, mAb 6G6), residues 223–242 (dark green, mAb 9C9), residues 230–239 (yellow, mAb 2C4), residues 278–292 (dark blue, mAb 10H10), residues 283–297 (brown, mAb 9C9), residues 288–302 (blue, mAb 6G6), residues 308–322 (magenta, mAb 9C9), residues 313–327 (purple, mAb 6G6), residues 398–412 (orange, mAb 6G6) and residues 403–417 (red, mAb 3E6). The sequence alignment was carried out using the program Muscle.

HtrA1 and HtrA3 (HtrA3-L) share a similar domain organization (Figure 1A) and 61% sequence identity [7], the sequence alignment is shown in Figure 2. The only available crystal structure of HtrA3 is that of the PDZ domain [44]. However, crystal structures of the IGFB, Kazal and protease domains of HtrA1 are available [27], [28] and these were used to model the corresponding HtrA3 domains. The sequence identity between the HtrA1 and HtrA3 IGFB, Kazal and protease domains is 53%, 53% and 76%, respectively [alignments were carried out using the program Muscle, (http://toolkit.tuebingen.mpg.de/muscle)]. The trimeric HtrA3 homology model was constructed in two stages: the first involved the construction of a monomer model and the second, packing three HtrA3 monomers to form a trimer.

The monomeric homology model of HtrA3 consisting of residues 24–453, without the signal peptide (residues 1–23), was constructed as follows. The HtrA1 and HtrA3 amino acid sequences for the (i) N-terminal region (i.e. the IGFB and Kazal domains) and (ii) protease domain were aligned using the default parameters in the program Muscle. The resulting alignment of the N-terminal regions was used to model HtrA3 residues 24–130 using the program Modeller v9.9 (http://toolkit.tuebingen.mpg.de/modeller#, [45]). The crystal structure of the N-terminal region (PDB id: 3TJQ, [27]) of human HtrA1 was the structural template for the HtrA3 N-terminal region (residues 24–130). Likewise, the homology model for HtrA3 residues 131–350 was constructed using the crystal structure of the catalytically active human HtrA1 protease domain (PDB id: 3NZI, A chain [28]) as the template. The SAXS envelope for full length HtrA1, kindly provided by Charles Eigenbrot and Mark Ultsch [27], was used to guide the manual placement of the HtrA3 N-terminal region in relation to the protease domain. Refinement of the orientation of the HtrA3 N-terminal region (residues 24–130) to the protease domain (residues 131–350) was then performed using the RosettaDock Server (Rosetta Suite 2.1; http://rosettadock.graylab.jhu.edu/). The top 10 solutions obtained from RosettaDock were overlaid onto the HtrA1 SAXS envelope and the one with the best fit was used in the HtrA3 homology model. The C-terminus of the HtrA3 N-terminal region (i.e. residue 130) was attached manually to the N-terminus (i.e. residue 131) of the HtrA3 protease domain. The missing residues (Pro-380 and Glu-381) in the crystal structure of the human HtrA3 PDZ domain (PDB id: 2P3W, A chain) were manually inserted. Using the HtrA1 SAXS envelope as a guide, HtrA3 residues Asp-351, Trp-352 and Lys-353 were manually added to the C-terminus of the HtrA3 protease domain and then the N-terminus of the HtrA3 PDZ domain was manually attached to Lys-353, ultimately generating the monomeric homology model of HtrA3.

To construct the trimeric HtrA3 homology model, the protease domain (residues 131–350) of the monomeric HtrA3 model was aligned to each of the protein chains in the trimeric HtrA1 protease domain crystal structure (PDB id: 3NZI, A, B and C chains [28]). The three HtrA3 monomers packed together with only a few minor steric conflicts between adjacent monomer side-chains; to alleviate these steric problems, the conformation of the side-chains were adjusted using amino acid conformer libraries. The HtrA3 monomeric and trimeric models were geometry optimized after each model building step for at least 2000 iterations (or until the gradient of successive iterations was <0.05 kcal/mol • Å) using the molecular mechanics Amber02 force field, Amber partial atomic charges and conjugate gradient minimization method (all other parameters were at default values) within the program Sybyl-X 2.0 (Certara, L.P.; http://tripos.com). All manual manipulations were performed using Sybyl-X 2.0. The resulting trimeric HtrA3 homology model was deemed to be a good quality model using PROCHECK [46], with 98.6% of residues in favored (89.8%) or allowed (8.8%) conformations.

### Statistics

Data are expressed as mean ± SEM of fold changes relative to control. Statistical analysis was performed on raw data using one-way ANOVA and Tukey’s post hoc test using PRISM version 5.00 (GraphPad Software, San Diego, CA), and P<0.05 was taken as significant.

## Results

### Epitopes of HtrA3 mAbs and their specificity

The domain organization of all four human HtrAs (1–4) is schematically illustrated in Figure 1A, and the similarities and differences between human HtrA3-L and HtrA3-S isoforms are shown in Figure 1B. Immunization of mice against a minimal mutant of HtrA3-L (HtrA3-L-S305A) or a synthetic peptide (residues 230–239 of human HtrA3-L) and subsequent cloning resulted in five distinct HtrA3 mAbs: 3E6, 6G6, 2C4, 10H10 and 9C9 [41]. The linear epitopes of these mAbs, schematically shown in Figure 1B, corresponded to the following residues in HtrA3: 3E6, 403–417; 6G6, 73–92, 133–147, 288–302, 313–327 and 398–412; 2C4, 230–239; 10H10, 278–292; and 9C9, 223–242, 283–297 and 308–322 (Figure 2).

All five mAbs were previously shown by Western blot to recognize wild type HtrA3 (both isoforms or HtrA3-L only), but not rHtrA1 or rHtrA2 [41]. While HtrA3-L was recognized by all five mAbs, HtrA3-S was not detected by 3E6 or 6G6, consistent with their epitopes containing HtrA3-L specific sequences in the PDZ domain (Figure 1B) [41]. In this study, we further confirmed that these mAbs were highly specific to HtrA3 against the entire human HtrA family including the newly discovered HtrA4 (Figure 1C). While rHtrA4 was detected by an HtrA4 antibody, none of the HtrA3 mAbs recognized HtrA4 (Figure 1C).

### Identification of HtrA3 activity modulating mAbs

**Figure 3:**
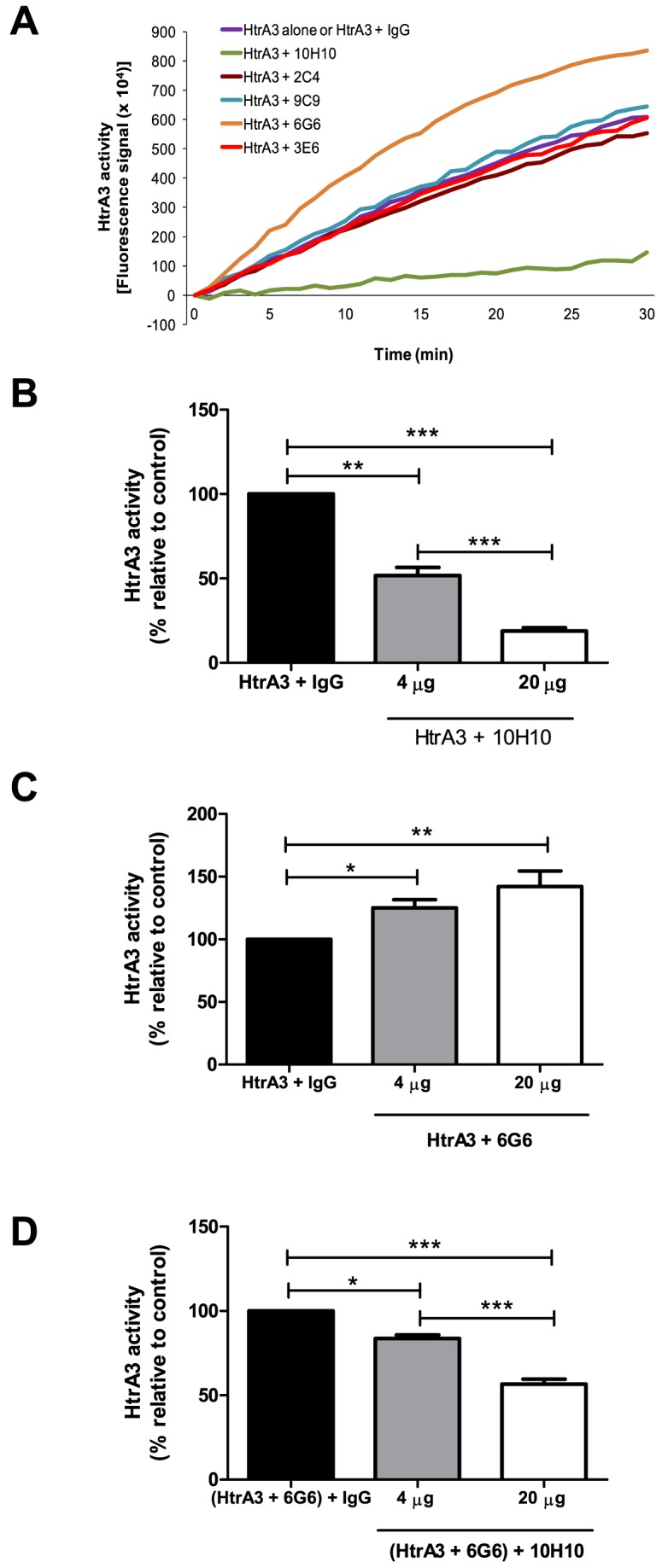
Effects of HtrA3 mAbs on substrate cleavage and dose-dependent modulation of HtrA3 activity by mAbs. (A) Representative real-time progressive curves of substrate cleavage by recombinant wild type human HtrA3 (HtrA3-L) in the presence of 20 µg/ml individual HtrA3 mAb (6G6, 9C9, 10H10, 3E6 and 2C4) or control mAb. (B) Inhibition of HtrA3 activity by mAb 10H10. (C) Enhancement of HtrA3 activity by mAb 6G6. (D) Inhibition of HtrA3 activity by mAb 10H10 subsequent to 6G6 stimulation. The data are expressed as changes in the rate of substrate cleavage relative to the control (B & C: control  =  HtrA3 with IgG control mAb, D: control  =  HtrA3 with mAb 6G6 at 20 µg/ml). Data are mean ± SEM from 3 independent experiments, *, P<0.05, **, P<0.01, ***, P<0.001.

We next assessed whether these HtrA3 mAbs could modulate the proteolytic activity of HtrA3. When pure human HtrA3 (HtrA3-L) was incubated with a fluorescence-quenched peptide substrate, a progressive increase in fluorescence signal resulting from substrate cleavage was detected (Figure 3A). To test the effects of the mAbs on HtrA3 activity, an equal amount of each individual HtrA3 mAbs or control IgG (20 µg/ml) was added into the enzyme reaction and the substrate cleavage kinetics were monitored. Compared to the control mAb, 10H10 reduced whereas 6G6 enhanced the proteolysis, while mAbs 3E6, 2C4 or 9C9 did not significantly affect the HtrA3 activity (Figure 3A). In the presence of mAb 6G6, the peptide substrate cleavage was faster (Figure 3A). These data indicate that mAb 6G6 stimulates whereas 10H10 inhibits HtrA3 activity.

To further investigate dose-dependent effect of mAbs 6G6 and 10H10, the enzyme reaction was carried out with different concentrations of these mAbs (0, 4 and 20 µg/ml). To illustrate the dose-dependency, the rate of substrate cleavage was expressed as a percentage of the control (containing control mAb at 4 or 20 µg/ml). Indeed, 10H10 inhibited (Figure 3B) whereas 6G6 stimulated (Figure 3C) HtrA3 activity in a clear dose-dependent manner. No enzyme activity was detected when 6G6 alone was incubated with the substrate, confirming that 6G6 itself had no peptidase activity (data not shown).

We next investigated whether HtrA3 activity, following mAb 6G6 stimulation, could be inhibited by mAb 10H10. HtrA3 was first incubated with 6G6 (20 µg/ml) and then with 10H10 (4 or 20 µg/ml) before the substrate cleavage was determined. The mAb 10H10 inhibited HtrA3 activity after 6G6 stimulation and the inhibition was also dose-dependent (Figure 3D), further confirming the blocking function of mAb 10H10. Likewise, we examined whether HtrA3, following 10H10 inhibition, could be activated by 6G6. However, no activity was detected when HtrA3 was first incubated with 10H10 (20 µg/ml) then with 6G6 (4 or 20 µg/ml, data not shown).

Neither the 10H10 nor 6G6 had any effect on the proteolytic activity of other human HtrA members – HtrA1, HtrA2 and HtrA4 (data not shown), consistent with these two HtrA3 mAbs recognizing HtrA3 only (Figure 1C).

### Confirmation that mAb 10H10 increases trophoblast migration and invasion *in vitro*

**Figure 4:**
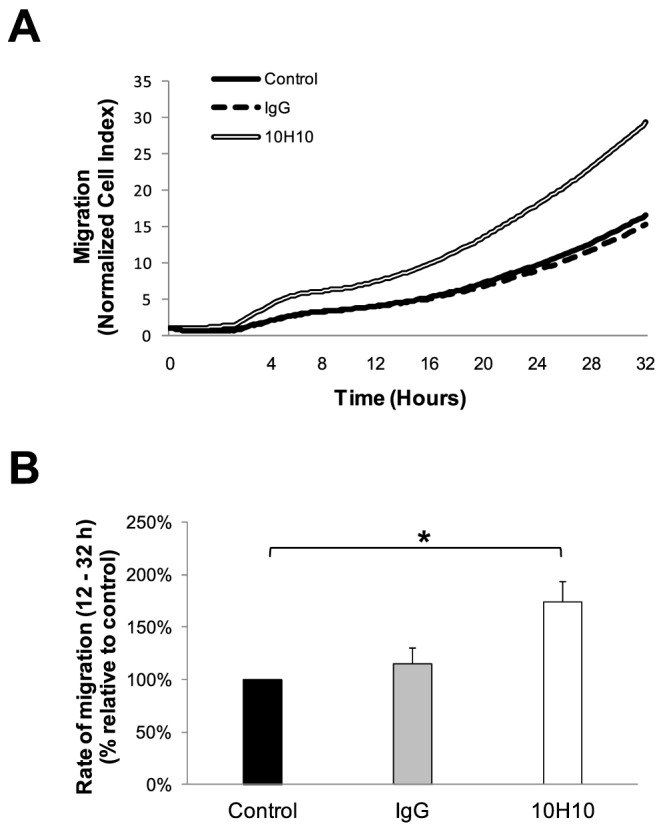
Enhancement of trophoblast migration by mAb 10H10. (A) Representative cell index curves of HTR8 cells for migration in the absence or presence of 10H10 or control mAb (5 µg/ml) measured with xCELLigence system. (B) Exogenous addition of mAb 10H10 (5 µg/ml) significantly increased HTR8 cell migration at 12–32 hours, compared to untreated controls. Data are mean ± SEM from 3 independent experiments, *, P<0.05.

**Figure 5:**
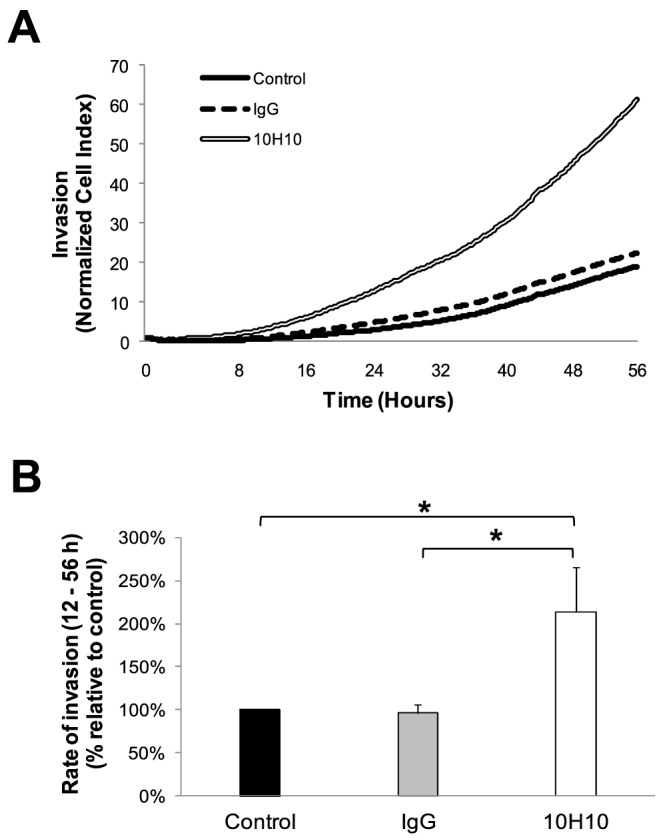
Enhancement of trophoblast invasion by mAb 10H10. (A) Representative cell index curves of HTR8 cells for invasion in the absence or presence of 10H10 or control mAb (5 µg/ml) measured with xCELLigence system. (B) Exogenous addition of mAb 10H10 (5 µg/ml) significantly increased HTR8 cell invasion at 12–56 hours, compared to untreated controls. Data are mean ± SEM from 3 independent experiments, *, P<0.05.

We next determined whether these HtrA3 activity-modulating mAbs would affect the function of HTR8 trophoblast cells expressing HtrA3 [33], [34]. Initially, real-time cell proliferation was monitored to assess the effects of these mAbs on cell growth and survival. Neither 10H10 nor 6G6 (both at 5 µg/ml) significantly altered cell growth (data not shown). However, 10H10 significantly increased the migration (Figure 4A & B) as well as invasion (Figure 5A & B) of HTR8 cells, confirming previous reports that HtrA3 inhibition enhances cellular migration and invasion without affecting growth [33], [34]. In contrast, when the experiment was repeated for mAb 6G6, no significant effects on cell migration or invasion were observed (data not shown).

### The 3-dimensional homology model of HtrA3

**Figure 6:**
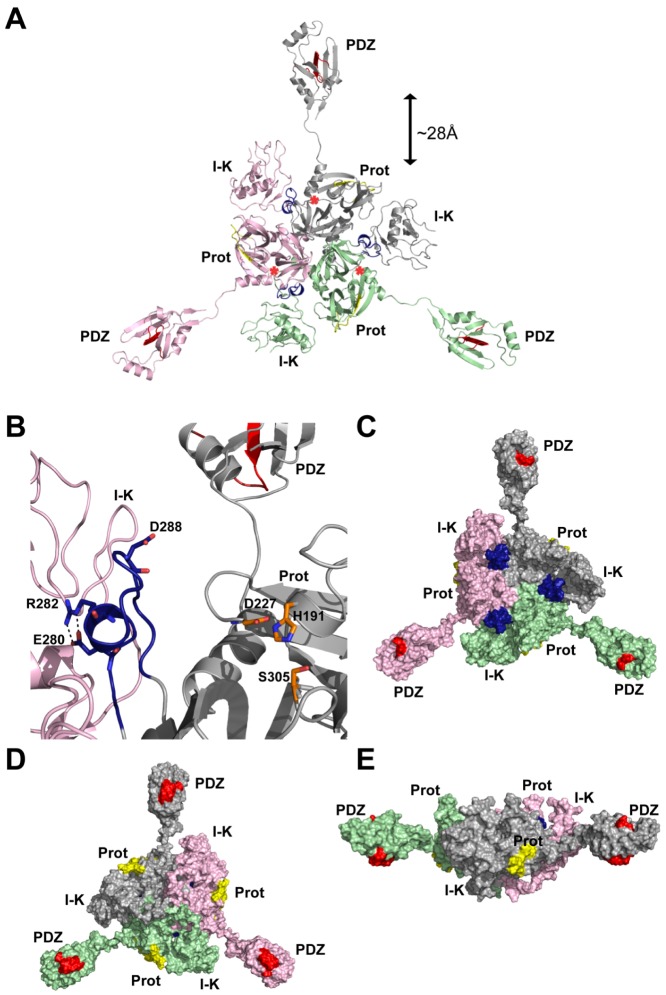
The trimeric HtrA3 homology model and location of the epitopes for mAbs 10H10, 2C4 and 3E6. (A) Cartoon representation of the trimeric HtrA3 (HtrA3-L) homology model, with each of the three HtrA3 monomers colored differently (grey, light green and light pink). Individual domains within each monomer are labeled: I-K; N-terminal combined IGFB-Kazal domains; Prot; central protease domain, and PDZ; the PDZ domain. Red asterix indicates the location of the catalytic site in each monomer. Epitope for the inhibitory mAb 10H10, residues 278–292 in the protease domain, is colored dark blue and corresponds to the putative HtrA3 sensor loop L3 [28]. Epitopes for mAbs 2C4 (residues 230–239) in each protease domain and 9C9 (residues 403–417) located in the PDZ domain are colored yellow and red, respectively. View directly above the protease catalytic sites. (B) Close up view of the 10H10 inhibitory epitope (dark blue) and modeled protease catalytic triad (orange sticks) for one HtrA3 monomer (grey cartoon). Catalytic triad residues His-191, Asp-227 and Ser-305 are displayed as orange sticks; Glu-280, Arg-282 and Asp-288 of the putative L3 sensor loop are displayed as dark blue sticks. Salt bridge/hydrogen bond interactions between Glu-280 and Arg-282 are shown as dashed black lines. The adjacent HtrA3 monomer is shown as a light pink cartoon. (C) Same view as in panel (A), protein is depicted as a molecular surface. (D) View of the non-catalytic face of HtrA3, i.e. a rotation of +180^o^ about the Y-axis from the view shown in panels (A) and (C). (E) Side view of the grey monomer [−90^o^ rotation about the X-axis, followed by a +120^o^ rotation about the Y-axis from the view shown in panel (A)]. One letter amino acid codes have been used for labels.

The trimeric human HtrA3 homology model shown in Figure 6A is structurally analogous to the full length human HtrA1 solution structure determined by Eigenbrot and coworkers [27] using SAXS. The overlay of the trimeric HtrA3 homology model with the HtrA1 SAXS envelope is shown in Figure S1. The full sequence comparison of the four human HtrA subtypes is given in Figure 2. The N-terminal IGFB-Kazal domains are connected to the HtrA3 protease domains by a flexible linker of ∼20 amino acids (designated Linker 1 in Figure 2) and are situated in between the protease domains of adjacent monomers (Figure 6A). Although a total of eight disulfide bridges can be predicted in the N-terminal IGFB-Kazal domains, only seven are observed in the crystal structure, one is missing due to structural disorder [27]. These disulfide bridges appear to be conserved in both HtrA3 and HtrA4 (the eight pairs of Cys residues involved in the HtrA1 disulfide bridges are identified in Figure 2 by paired numbers 1-1’, 2-2’ and so on above the HtrA3 sequence). In the HtrA3 model, the sixteen Cys residues are able to form equivalent disulfide bridges. Interestingly, the other domains of HtrA1, HtrA3 and HtrA4 do not contain any Cys residues. The N-terminal domain of HtrA2 has a different architecture to that of the other three HtrA subtypes (Figure 1A) and it does not contain the same Cys residue pattern (Figure 2).

The protease domain of the HtrA family is structurally conserved and adopts a canonical trypsin fold consisting of two β-barrels with a couple of α-helices attached [27], [28]. The catalytic Ser-His-Asp triad is located in between the two β-barrels. The HtrA3 protease catalytic Ser residue has been determined by mutagenesis to be Ser-305 and is contained within the mammalian protease GNSGGPL sequence motif [7], [41], [42]. Likewise, the catalytic HtrA3 His-191 residue has been identified by the mammalian HtrA protease TNAHV sequence motif. The putative catalytic Asp-227 residue was identified by analogy with HtrA1 and HtrA2 from sequence alignments (Figure 2) [7], [41]. The location of the catalytic site in each of the three modeled HtrA3 protease domains is indicated by the red asterisks in Figure 6A and the catalytic triad residues His-191, Asp-227 and Ser-305 are highlighted in Figure 6B. In HtrA1 the sensor loop L3 (defined in Figure 2) is located on the catalytic face of the protease domain and interacts directly with the enzyme substrate. This is a key step in the activation of the HtrA1 protease [28]. The distance between the HtrA1 L3 sensor loop and the adjacent catalytic site is ∼10 Å. Due to the structural conservation of the HtrA protease domain and the sequence similarity between HtrA1 and HtrA3, we would expect the HtrA3 L3 sensor loop (corresponding to residues 280–293, Figure 2) and catalytic triad to be a similar distance apart.

**Figure 7:**
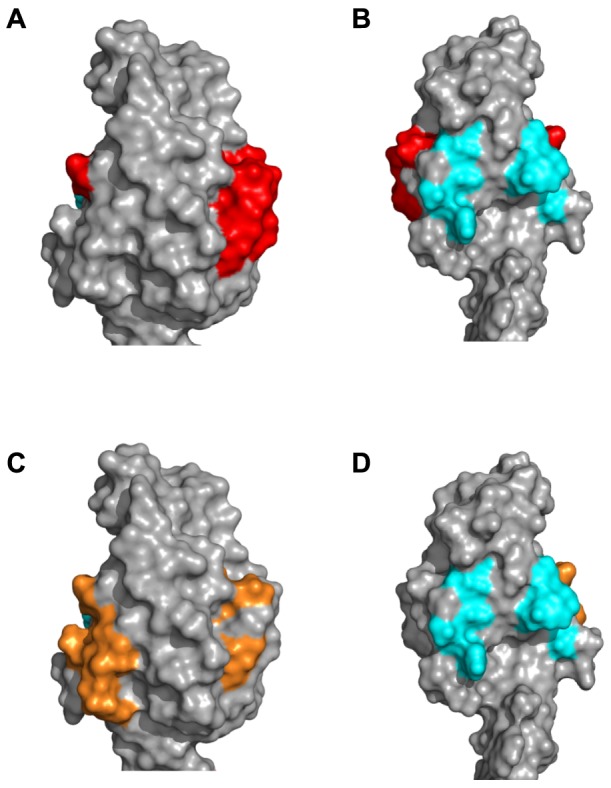
Comparison of the PDZ domain epitopes for mAbs 3E6 and 6G6. The PDZ domain for the grey monomer is shown as a molecular surface. (A) The 3E6 (residues 403-417) epitope is colored red. (B) View is a +180^o^ rotation about the Y-axis from the view in panel (A). The light blue patches are the HtrA3 residues (Arg-360, Thr-363, Gln-389, Glu-390, Ala-392, Ser-419, Ser-420 and Gln-423) involved in protein/peptide interactions. (C) Same view as in panel (A), the 6G6 epitope corresponding to residues 398-412 is colored orange. (D) The 6G6 epitope, same view as in panel (B).

The C-terminal HtrA3 PDZ domain, is a protein-protein interaction domain, is comprised of five β-strands which form a β-sandwich structure and three α-helices. The residues involved in protein or peptide binding are Arg-360, Thr-363, Gln-389, Glu-390, Ala-392, Ser-419, Ser-420 and Gln-423 [colored light blue in Figure 7B & D, [44]]. The PDZ domains are connected to the HtrA3 protease domains by a flexible linker (∼11 amino acids, designated Linker 2 in Figure 2), and are located ∼28 Å distant from the protease domains (Figure 6A).

Although the trimeric HtrA3 homology model fits the HtrA1 SAXS envelope extremely well, the N-terminal region, the protease domain and the PDZ domain could adopt alternative packing arrangements in solution due to the flexible linkers connecting them. It has also been reported that the N-terminal and PDZ domains of HtrA1 are not required for protease activity [27]–[29]. Previous studies have confirmed that HtrA3-S lacking the PDZ domain is also proteolytically active [34], [42]. Whether the N-terminal of HtrA3 is required for protease activity remains to be determined.

### Mapping the five mAb epitopes onto the HtrA3 model

**Figure 8:**
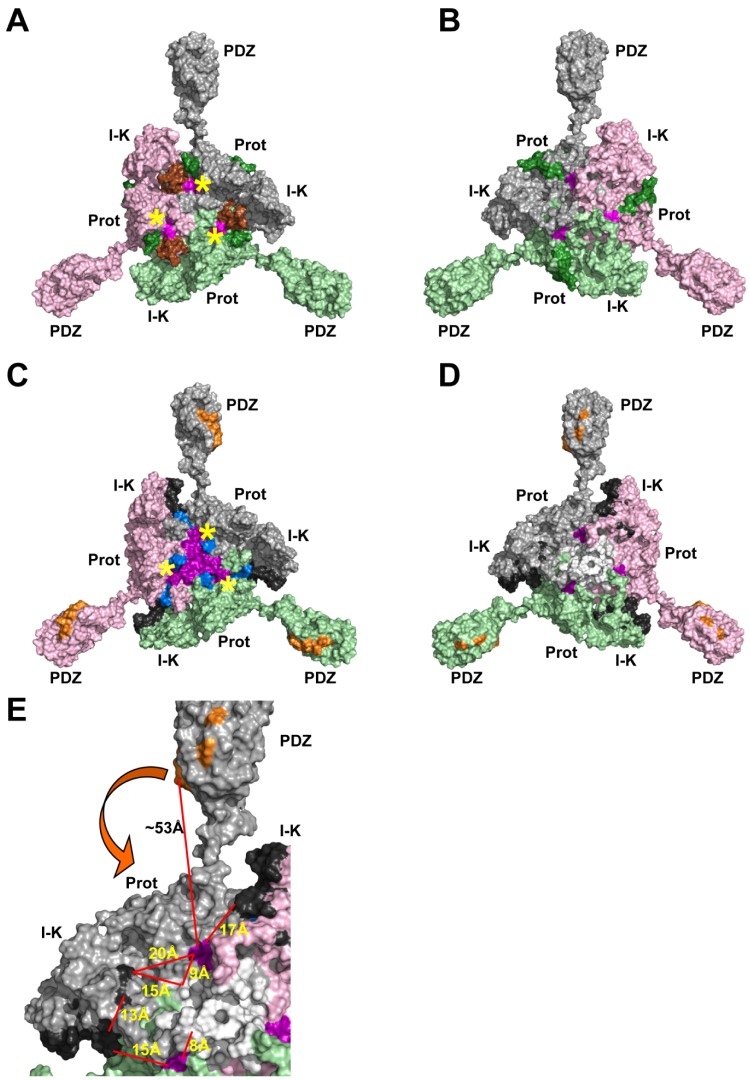
Location of the epitopes for mAbs 9C9 and 6G6. (A) Same view as in Figure 6A & C. The epitope regions for mAb 9C9 are shown: residues 283–297 (colored brown), residues 224–227 (from the 223–242 peptide, colored dark green) and residues 320–322 (from the 308–322 peptide, colored magenta). (B) A +180^o^ rotation about the Y-axis from view shown in panel (A), showing the non-catalytic face of HtrA3. Residues 228–242 are colored dark green and 308–319 are colored magenta. (C) The epitope regions for mAb 6G6 are shown: residues 73–78 (from the 73–92 peptide, colored dark grey), residues 288–302 (colored blue), residues 322–327 (from the 313–327 peptide, colored purple), and residues 398–412 (colored orange). (D) A +180^o^ rotation about the Y-axis from view shown in panel (C), showing the non-catalytic face of HtrA3. Residues 79–92 are colored dark grey and 313–321 are colored purple. The 6G6 epitope residues 133–147 (colored white) are now visible on the protease domain surface. (E) Close up view of panel (D) showing the 6G6 epitope regions on the non-catalytic face of HtrA3. Distances between some of the epitope regions are shown in Å. The arrow indicates the direction the PDZ domain would need to move to place the 6G6 epitope residues 398–412 (colored orange) in closer to the residues 313–321 (colored purple), 133–147 (colored white) and 79–92 (colored dark grey). In panels (A) and (C), the location of the catalytic site in each monomer is indicated by a yellow asterix.

Antibody epitopes can be either continuous or discontinuous on the target protein surface. Mapping the location of antibody epitopes by screening linear peptide libraries is a common initial approach adopted by both academia and Big Pharma [47], [48]. If there is sequence or shape similarity between peptides in the library then false positives can arise using this mapping. The linear epitopes, determined by peptide library screening for each of the five HtrA3-specific mAbs (Figure 1B & 2) [41], were mapped onto the 3D HtrA3 model (Figure 6–8) to visualize their location and to understand how they may modulate protease activity. Antibodies 10H10, 2C4 and 3E6 have continuous epitopes whereas mAbs 9C9 and 6G6 appear to have discontinuous epitopes.

The putative HtrA3 L3 sensor loop (residues 280–293, Figure 2) corresponds to the epitope of the inhibitory mAb 10H10 (residues 278–292; colored dark blue in Figure 2 & 6A-C). An antibody binding to residues 278–292 would not only prevent a substrate from interacting with the L3 sensor loop, but also block its access to the catalytic triad, thereby inhibiting the protease activity of HtrA3. Such a mode of action is consistent with 10H10 being a neutralizing mAb (Figure 3).

HtrA3 mAb 2C4 was raised against a single peptide corresponding to residues 230–239 (colored yellow in Figure 2, 6A, D & E). In the HtrA3 homology model, residues 230–239 lie on an outer surface loop of the protease domain on the opposite face to the catalytic site (Figure 6D & E) and would be accessible by an antibody without impacting on the catalytic residues. The location of the 2C4 epitope is consistent with this mAb having no impact on the *in vitro* enzyme activity of HtrA3 (Figure 3A).

The mAb 3E6 was shown by Western blot to recognize HtrA3-L but not the short isoform (HtrA3-S) [41], hence the PDZ domain appears to be required for the binding of this mAb. The epitope for mAb 3E6, corresponding to residues 403–417 (colored red in Figure 2, 6A-E, 7A & B), lies solely in the PDZ domain and is largely surface exposed. It is located on the opposite surface to the PDZ domain protein/peptide binding groove (colored light blue in Figure 7B). An antibody would be able to bind to this epitope without blocking access to the PDZ domain protein/peptide binding groove and would also have no direct contact with the protease domain or its catalytic site when the PDZ domain is extended ∼28 Å out from the central protease domain. This is highly consistent with the HtrA3 activity data (Figure 3A). The lack of any influence by mAb 3E6 on protease activity is also in accordance with the observation that the HtrA1 PDZ domain does not have any involvement in protease activity or enzyme regulation [27], [28].

Like 2C4 and 3E6, mAb 9C9 also has no effect upon HtrA3 protease activity (Figure 3A); but it appears to have a discontinuous epitope as three linear peptides in the library screen displayed affinity for this mAb. The three linear peptides correspond to HtrA3 protease domain residues 223–242, 283–297 and 308–322 (colored dark green, brown and magenta, respectively in Figure 2, 8A & B). Residues 283–297 (colored brown in Figure 2 & 8A) are located on the catalytic face of HtrA3 and encompass the majority of the putative sensory loop L3 (residues 280–293). In addition, residues 224–227 (four residues in the epitope region 223–242) and 320–322 (three residues in the epitope region 308–322) are located on the protease catalytic face (dark green and magenta patches, respectively, located next to the brown patches in Figure 8A). If these residues (224–227, 283–297 and 320–322) were part of the 9C9 epitope, then this mAb would be expected to inhibit HtrA3 activity, in a similar manner to mAb 10H10, but this is not the case (Figure 3A). There is a 53% sequence similarity between the linear peptides corresponding to residues 283–297 and 308–322. Given that mAb 9C9 has no effect upon HtrA3 enzyme activity, we propose that residues 283–297 are not part of the 9C9 epitope but a false positive of the peptide library mapping assay. The region corresponding to residues 223–242 (dark green in Figure 2, 8A & B) encompasses the epitope of mAb 2C4 (residues 230–239, colored yellow in Figure 2, 6A, D & E). Residues 308–322 (colored magenta in Figure 2, 8A & B) are located on two β-strands and their connecting loop. Visual inspection of these two regions in the HtrA3 model showed that residues 228–242 from the 223–242 peptide and residues 308–319 from the 308–322 peptide are surface exposed and located on the non-catalytic face of the protease domain (colored dark green and magenta, respectively in Figure 8B). In each monomer these two regions would be accessible by a single antibody and their location on the HtrA3 non-catalytic face is consistent with the 9C9 mAb having no effect upon protease activity.

In contrast to mAb 10H10, 6G6 stimulates HtrA3 protease activity (Figure 3). The discontinuous 6G6 epitope identified by the linear peptide library screen corresponds to residues 73–92 (IGFB domain, colored dark grey in Figure 2 & 8C-E), 133–147 (Kazal domain and linker to the protease domain, colored black in Figure 2 and white in Figure 8D & E), 288–302 (protease domain, colored blue in Figure 2 & 8C), 313–327 (protease domain, colored purple in Figure 2 & 8C-E) and 398–412 (PDZ domain, colored orange in Figure 2, 7C, D & 8C-E). Residues 288–302 (colored blue in Figure 2 & 8C) cover the majority of the putative HtrA3 L3 sensor loop (residues 280–293) located on the protease domain catalytic face. Antibody interaction with residues 288–302 would be expected to block HtrA3 enzyme activity; however, mAb 6G6 acts in the opposite manner and stimulates HtrA3 enzyme activity (Figure 3). HtrA3 residues 288–302 has 53% sequence similarity to residues 313–327 (colored purple in Figure 2 & 8C-E) and we therefore propose that the linear peptide corresponding to HtrA3 residues 288–302 is a false positive in the epitope mapping assay. Although residues 73–78 (six residues in the epitope region 73–92) and 322–327 (six residues in the epitope region 313–327) are located on the protease catalytic face (dark grey and purple patches, respectively, located in the IGFB and protease domains near the blue patches in Figure 8C), the remainder of these two epitopes (i. e. residues 79–92 and 313–321) are located on the opposite face of HtrA3 (Figure 8D). Residues 79–92 (from epitope peptide 73–92), 313–321 (from epitope peptide 313–327) and 133–147 are located in close proximity to each other on the opposite face of HtrA3 to the protease catalytic sites (colored dark grey, purple and white, respectively, in Figure 8D & E). Distances between some of these epitope regions are shown in Figure 8E. Located>53 Å from these three regions in the HtrA3 model, the fifth linear peptide identified by mAb 6G6 corresponds to residues 398–412 in the PDZ domain (colored orange in Figure 7C, D & 8C-E) and overlaps with the linear peptide identified by mAb 3E6 (corresponding to residues 403–417, colored red in Figure 7A & B). How mAb 6G6 stimulates HtrA3 proteolytic activity is not immediately clear from the homology model. Under non-denaturing conditions, HtrA3-L was recognized by mAb 6G6 whereas HtrA3-S was not [41]; consistent with the Western blot data [41] and indicating that the PDZ domain is part of the mAb epitope. It is unlikely that all four regions corresponding to residues 73–92, 133–147, 288–302 and 313–327 are false positives of the linear peptide epitope mapping assay (although we do propose that 288–302 is a false positive). These data suggest two scenarios: (1) residues 398–412 in the PDZ domain alone are responsible for the stimulatory activity of mAb 6G6 or (2) all four regions (residues 73–92, 133–147, 313–327 and 398–412) are required for mAb 6G6 binding. The first scenario seems unlikely given the similarity to the mAb 3E6 epitope (compare Figure 7A-D). The second scenario is more probable, but a conformational change would be required to relocate the PDZ domain closer to the protease domain and the other epitope regions (i. e. movement in the direction of the arrow in Figure 8E).

Discontinuous antibody epitopes with extensive antigen interfaces have been previously reported, one prime example is the influenza virus N9 neuraminidase-NC41 Fab complex [49]. The interface between N9 neuraminidase and Fab NC41 buries 1815 Å^2^ of surface area and the three extremities of the triangle-shaped interface are 27 Å, 30 Å and 27 Å apart. The discontinuous Fab NC41 epitope on the N9 neuraminidase surface consists of nineteen residues located in five separate segments. The tertiary structure places a number of short loops in close proximity on the N9 neuraminidase surface and these form the discontinuous epitope; a scenario not dissimilar to the one proposed here for mAb 6G6. The distances between the mAb 6G6 epitope regions on the non-catalytic face of HtrA3 (Figure 8E) are well within the interface dimensions reported for the N9 neuraminidase, although the PDZ domain would need to move closer to the protease domain to bring the PDZ epitope region within 15–25 Å of the other epitope regions.

All five mAbs are highly specific to HtrA3 and did not detect human HtrA1, HtrA2 [41] or HtrA4 (Figure 1C). Sequence comparison of the HtrA3 mAb epitope regions within the human HtrA family members are shown in Figure 2. The 10H10 epitope (HtrA3 residues 278–292) in HtrA3 encompasses almost the entire L3 sensor loop. Allowing for conservative amino acid substitution there are only two, four and one residues in the L3 sensor loop of HtrA1, HtrA2, and HtrA4 respectively that differ from HtrA3 (Figure 2). Of these, HtrA3 Glu-280 interacts with Arg-282 to maintain a short α-helical segment (Figure 6B). Glu-280 in HtrA3 is replaced by Gly-303, Pro-281 and Gly-301 in HtrA1, HtrA2 and HtrA4 respectively, thereby removing the possibility of an interaction with Lys-305 (HtrA1), Arg-283 (HtrA2) or Lys-303 (HtrA4) to maintain the α-helical conformation observed in the HtrA3 model. It is thus possible that subtle conformational differences in the L3 sensor loop are responsible for the specificity of mAb 10H10 to HtrA3.

The stimulating mAb 6G6 has a discontinuous epitope including residues from the HtrA3 IGFB, Kazal, protease and PDZ domains (Figures 1B & 2). As the N-terminal architecture of HtrA2 is very different to that of HtrA1, HtrA3 or HtrA4 [7], it is not surprising that mAb 6G6 does not recognize HtrA2. The mAb 6G6 epitope region 313–327 is conserved amongst HtrA family members, therefore specificity of 6G6 for HtrA3 over HtrA1 and HtrA4 is most likely imparted by sequence differences with HtrA3 residues 73–92, 133–147 and 398–412 (Figure 2). The HtrA3 specificity of mAb 9C9 is most likely imparted by residues 223–242 as the epitope region 308–322 is highly conserved amongst the human HtrA subtypes. For mAbs 2C4 (HtrA3 residues 230–239) and 3E6 (HtrA3 residues 403–417) the sequence differences among the four human HtrA family members are likely to be responsible for HtrA3 specificity (Figure 2).

## Discussion

HtrA proteases have been implicated in the pathogenesis of several diseases such as cancers, neurodegenerative disorders and arthritis. However, there are no drugs available to treat diseases involving HtrA dysregulation. In this study, we characterized five highly specific HtrA3 mAbs and demonstrated that two of these specifically modulated (one inhibited and other stimulated) HtrA3 activity, providing unique research tools to investigate the molecular functions of HtrA3. The inhibitory HtrA3 mAb was further confirmed to increase trophoblast HTR8 cell migration and invasion in culture, demonstrating the potential therapeutic applications of this mAb to treat diseases associated with HtrA3 dysregulation. Furthermore, guided by published HtrA1 SAXS data [27], we constructed a 3D HtrA3 homology model to visualize the location of the mAb epitopes and to gain an understanding of their mechanism of action. Since mammalian HtrA1 and HtrA4 share similar domain organizations to that of HtrA3, the knowledge gained in this study about regulating HtrA3 activity may have boarder applications.

Western blot confirmed that all five HtrA3 mAbs were highly specific to HtrA3; none recognized any other human HtrA family members (HtrA1, HtrA2 or HtrA4). Among these five mAbs, 10H10 inhibited whereas 6G6 stimulated the proteolytic activity of HtrA3 in an *in vitro* protease activity assay. The differential effects of these two activity-modulating antibodies are striking. Together they provide us with highly specific tools to investigate HtrA3 cellular functions.

It was further confirmed by *in vitro* cell-based experiments that modulating the activity of HtrA3 by mAbs 10H10 or 6G6 had no effect on the growth of HTR8 trophoblast cells. The migration and invasion of HTR8 cells was significantly increased by the inhibitory mAb 10H10, consistent with our previous findings that HtrA3 is a negative regulator of invasion [33], [34]. The HtrA3 homology model offers an explanation as to why mAb 10H10 inhibits HtrA3 activity. The mAb epitope is located near the HtrA3 protease catalytic site and binding of the mAb would be expected to block substrate access to the catalytic site. The homology model was also able to suggest why mAbs 2C4 and 3E6 had no effect upon the proteolytic activity of HtrA3; their epitopes do not impact upon the protease domain catalytic site. Given that mAbs 9C9 and 6G6 do not inhibit protease activity like mAb 10H10, we propose that the peptides corresponding to HtrA3 residues 293–297 (mAb 9C9) and 288–302 (mAb 6G6) are false positives arising from the *in vitro* linear peptide library screen.

The epitope of mAb 9C9 would appear to be discontinuous and consist of two separate regions, residues 228–242 from the 223–242 peptide and 308–319 from the 308–322 peptide. The HtrA3 homology model indicates that neither region would impact on the HtrA3 catalytic site, consistent with mAb 9C9 having no affect upon protease activity. The mAb 6G6 epitope also appears to be discontinuous and involve all four domains of HtrA3: IGFB, Kazal, protease and PDZ domains. As 6G6 recognizes only the HtrA3-L isoform, its epitope region in the PDZ domain (residues 398–412) would appear to be crucial for mAb binding. The mechanism by which mAb 6G6 stimulates HtrA3 enzyme activity is not clear from the homology model. Three regions of the discontinuous 6G6 epitope corresponding to residues 79–92 (from the 73–92 peptide, located in the IGFB domain), 133–147 (Kazal domain) and 313–321 (from the 313–327 peptide, located in the protease domain) are located within close proximity to each other on the opposite face of HtrA3 to the protease catalytic sites (Figure 8E). However, the epitope region in the PDZ domain is>53 Å distant from these three regions. While it may be possible for an antibody to span such a large distance, it is more likely that there is a domain rearrangement bringing the PDZ domain closer to the other three epitope regions (indicated by the arrow in Figure 8E). It has been postulated by Truebestein and coworkers [28] that although the PDZ domain of human HtrA1 is not involved in enzyme activation, it may play a role in substrate processing by holding the protein/peptide substrate for C-terminal cleavage in the protease catalytic site. The mAb 6G6 may facilitate a more efficient substrate-holding process; consistent with our experimental observation that mAb 6G6 in a dose-dependent manner increased substrate cleavage. Future crystallographic and mutagenesis studies will establish how mAbs 6G6 and 9C9 interact with HtrA3 and confirm the blocking mechanism of mAb 10H10.

For HtrA1, it is thought that substrate-induced remodeling alters the conformation of the catalytic site loops, leading to enzyme activation. It has also been suggested that the catalytic site loops of HtrA1 undergo a disorder-to-order transition yielding a stably folded activation domain [28]. However, the structural rearrangement which occurs in the presence of the mAb 6G6 to possibly produce an activated form of HtrA3 is yet to be experimentally determined. HtrA protease substrate-induced activation may also coincide with conversion from lower to higher order oligomers [3]. Truebestein and coworkers [28] demonstrated that denatured citrate synthase as a substrate causes HtrA1 protease activation by stabilizing a higher-order multimer in a PDZ-independent manner. The possibility that HtrA3 may form higher order oligomers requires further biochemical and structural investigation, but HtrA3 is predicted to exist at least in the trimeric form. N-terminal aromatic residues in the modeled HtrA3 protease domain (Phe-140, Phe-142 and Phe-255) are important for mediating an intermolecular oligomerization network that forms a ring of π-π interactions to stabilize the trimer, analogous to the HtrA1 crystal structures [28]. To date, no full length 3D structure of HtrA1 or HtrA3 has been determined crystallographically. Since the epitope of mAb 6G6 appears to involve all four domains, it may prove to be a useful tool in the structural determination of HtrA3-L and allow the characterization of rearrangements that may occur upon trimerization and substrate binding at the HtrA3 protease domain.

Since HtrA3 is downregulated in a number of cancers, it is proposed to be a tumor suppressor [10], [37]–[39]. Previous studies have shown that HtrA1 is upregulated and activated during chemotherapy-induced cytotoxicity [50]. In addition, HtrA3 attenuates cell survival with either etoposide or cisplatin treatment in a manner dependent on serine protease function [10]. Thus, we tested whether stimulating HtrA3 activity with mAb 6G6 would modulate cell growth through a mechanism involving irreversible proteolysis of factors crucial to cell survival. However, our *in vitro* experiments with trophoblast HTR8 cells did not show a potential functional effect of mAb 6G6 on cell growth, migration or invasion, and the reasons for this require further investigation.

Various studies have demonstrated the utility of antibodies in treating human diseases such as transplant rejections, cancers, rheumatoid arthritis, Crohn’s disease and antiviral prophylaxis [reviewed in [51]]. Modulating HtrA3 activity using 6G6 or 10H10 in pathological conditions such as preeclampsia, arthritis and tumor progression [1], [10], [11], [15], [16] would provide novel therapeutic applications. HtrA3 is expressed and secreted by a wide range of tissues although at different levels [7]. Placental HtrA3 is secreted into the maternal circulation and its serum levels are higher during early pregnancy in women who later develop preeclampsia [15], [41]. Hence regulating HtrA3 activity with the inhibitory mAb 10H10 may provide a potential therapeutic opportunity in treating diseases where HtrA3 is abnormally high.

In summary, our study characterized the binding properties of five highly specific HtrA3 mAbs and identified that two of these modulated HtrA3 activity. These mAbs will provide useful tools to investigate the functional importance of HtrA3. They will also provide opportunities to characterize the effects of HtrA3 dysregulation in a number of diseases and cellular processes. Owing to the similarities in domain architecture, the knowledge gained in targeting HtrA3 may also have relevance to mammalian HtrA1 and HtrA4.

## Supporting Information

Figure S1 Comparison of the trimeric HtrA3 homology model with the HtrA1 SAXS envelope. (A) Cartoon representation of the trimeric HtrA3 homology model. Each of the monomers has been colored differently. The locations of the IGFB-Kazal (I-K), protease (Prot) and PDZ domains are indicated. The three catalytic sites are identified by yellow asterisks. View is looking directly down onto the catalytic face of the protease domains. (B) Side view of the HtrA3 homology model, i.e. view is a −90^0^ rotation about the X-axis from panel (A). (C) & (D) Overlay of the HtrA1 SAXS envelope (transparent light grey surface) onto the trimeric HtrA3 homology model and view as in panels (A) & (B), respectively.
(EPS)
